# Chemotherapy-Induced Peripheral Neuropathy Detection via a Smartphone App: Cross-sectional Pilot Study

**DOI:** 10.2196/27502

**Published:** 2021-07-05

**Authors:** Ciao-Sin Chen, Judith Kim, Noemi Garg, Harsha Guntupalli, Reshma Jagsi, Jennifer J Griggs, Michael Sabel, Michael P Dorsch, Brian C Callaghan, Daniel L Hertz

**Affiliations:** 1 Department of Clinical Pharmacy University of Michigan College of Pharmacy Ann Arbor, MI United States; 2 Department of Radiation Oncology University of Michigan Medical School Ann Arbor, MI United States; 3 Department of Internal Medicine Hematology & Oncology Division University of Michigan Medical School Ann Arbor, MI United States; 4 Department of Surgery University of Michigan Medical School Ann Arbor, MI United States; 5 Department of Neurology University of Michigan Medical School Ann Arbor, MI United States

**Keywords:** chemotherapy-induced peripheral neuropathy, smartphone, mobile health, gait, balance, 9-Hole Peg Test

## Abstract

**Background:**

Severe chemotherapy-induced peripheral neuropathy (CIPN) can cause long-term dysfunction of the hands and feet, interfere with activities of daily living, and diminish the quality of life. Monitoring to identify CIPN and adjust treatment before it progressing to a life-altering severity relies on patients self-reporting subjective symptoms to their clinical team. Objective assessment is not a standard component of CIPN monitoring due to the requirement for specially trained health care professionals and equipment. Smartphone apps have the potential to conveniently collect both subjective and objective CIPN data directly from patients, which could improve CIPN monitoring.

**Objective:**

The objective of this cross-sectional pilot study was to assess the feasibility of functional CIPN assessment via a smartphone app in patients with cancer that have received neurotoxic chemotherapy.

**Methods:**

A total of 26 patients who had completed neurotoxic chemotherapy were enrolled and classified as CIPN cases (n=17) or controls (n=9) based on self-report symptoms. All participants completed CIPN assessments within the NeuroDetect app a single time, including patient-reported surveys (CIPN20 [European Organization for Research and Treatment of Cancer Quality of Life Questionnaire for Chemotherapy-induced Peripheral Neuropathy 20-item scale] and PRO-CTCAE [Patient-Reported Outcomes version of the Common Terminology Criteria for Adverse Events]) and functional assessments (Gait and Balance and 9-Hole Peg Test). Functional assessment data were decomposed into features. The primary analysis was done to identify features indicative of the difference between CIPN cases and controls using partial least squares analyses. Exploratory analyses were performed to test if any features were associated with specific symptom subtypes or patient-reported survey scores. Patient interviews were also conducted to understand the challenges they experienced with the app.

**Results:**

Comparisons between CIPN cases and controls indicate that CIPN cases had shorter step length (*P*=.007), unique swaying acceleration patterns during a walking task, and shorter hand moving distance in the dominant hands during a manual dexterity task (variable importance in projection scores ≥2). Exploratory analyses showed similar signatures associated with symptoms subtypes, CIPN20, and PRO-CTCAE. The interview results showed that some patients had difficulties due to technical issues, which indicated a need for additional training or oversight during the initial app download.

**Conclusions:**

Our results supported the feasibility of remote CIPN assessment via a smartphone app and suggested that functional assessments may indicate CIPN manifestations in the hands and feet. Additional work is needed to determine which functional assessments are most indicative of CIPN and could be used for CIPN monitoring within clinical care.

## Introduction

Up to 70% of patients receiving neurotoxic chemotherapy experience chemotherapy-induced peripheral neuropathy (CIPN) [1]. CIPN manifests in the feet or hands primarily as numbness or tingling, but it can also have motor or painful components [2,3]. In some patients, CIPN is irreversible, causing long-term interference with balance and dexterity, increased risk of falls [4], and negative effects on quality of life [5].

After neurotoxic chemotherapy, patients self-report symptoms of CIPN during regular appointments with their medical oncology team [6]. Patients who report CIPN symptoms may undergo objective assessment, but this is not standard of care due to the requirement for specially trained health care professionals and equipment [7]. There have been recent efforts to develop validated patient-reported outcome (PRO) questionnaires for CIPN assessment, including the European Organization for Research and Treatment of Cancer Quality of Life Questionnaire for Chemotherapy-induced Peripheral Neuropathy 20-item scale (CIPN20) [8]. PROs are an improvement over routine clinician assessment but are limited by a lack of objective assessment and the complete reliance on patients’ willingness and ability to accurately report CIPN [9].

Objective evidence of neuropathy includes gait impairment and increased sway, which can be detected by wearable sensors [10,11]. Another common manifestation is reduced manual strength and dexterity, which can be assessed using functional testing with a 9-Hole Peg [12]. Collecting PRO during chemotherapy via a smartphone app has been integrated into clinical practice to monitor and reduce severe toxicity [13]. Smartphone apps that integrate PRO and objective assessment could improve CIPN detection with minimal inconvenience and cost to the patient or health care system [14]. The objectives of this pilot study were to assess the feasibility of patients downloading and completing objective CIPN assessments remotely within a smartphone app and to explore whether there were differences in the objective functional data between patients with and without CIPN.

## Methods

### Study Patients

Patients with cancer aged over 18 who had completed neurotoxic chemotherapy including a taxane, platinum, or vinca alkaloid and had access to an iPhone were enrolled in this cross-sectional study. All patients completed written informed consent. This study was approved by the University of Michigan Institutional Review Board (IRBMed).

### Completion of NeuroDetect

All enrolled patients were emailed instructions on how to download NeuroDetect version 1.0 (available in the iOS app store) and were asked to complete all data collection assessments within the app a single time. Data collection included a baseline demographic survey, patient-reported CIPN assessment, and 2 functional CIPN assessments (the Gait and Balance and 9-Hole Peg Tests) available within Apple’s ResearchKit.

### Baseline Demographics

Patients self-reported standard demographics and information about their cancer, including date of diagnosis, cancer type, chemotherapy agents, whether they had surgery, surgery date, and if they were taking pain medication.

### Patient-Reported CIPN

The CIPN20 is considered the gold standard for CIPN assessment [15]. CIPN20 asks patients to indicate the degree of 20 CIPN symptoms they experienced within the past week on a numerical scale from 1 to 4 (1=not at all, 2=a little, 3=quite a bit, and 4=very much). CIPN20 includes 9 sensory items, 8 motor items, and 3 autonomic items [8]. In exploratory analyses, to test if any features that are specifically associated with CIPN cases having specific types of symptoms, we created subscales including the first 8 sensory symptoms items (CIPN8) [16], the first 4 sensory symptoms items (CIPN4), the first 2 feet items (CIPN2Feet), the first 2 hands items (CIPN2Hands), and all 8 motor items (CIPNM) as summary indicators in this exploratory study. CIPN20 scores and all subscales were linearly converted to 0-100 [8].

In addition, patient-reported CIPN was collected via PRO-CTCAE, which collects the severity and interference of neuropathy symptoms [17]. Patients also completed a falls diary, which asked whether or not the patient has fallen since the last chemotherapy session [18,19].

### Functional CIPN Analysis

In the Gait and Balance test, patients were asked to walk 7 steps in a straight line, turn around, and walk back to where they started with their smartphone in a pocket or bag. Walking forward and walking back were treated as 2 replications. User data were collected by the inertial accelerometer and pedometer and processed by iOS’s sensor fusion algorithm [20]. Vertical acceleration data were obtained by rotating user acceleration with orientation information. Horizontal acceleration data were obtained by assuming the axis with a larger SD of the velocity, which was calculated as the sum of the integral of acceleration, was the moving direction of the patient. Horizontal acceleration data from patients who did not follow instructions to put their phone in their pocket and instead held their phone in their hands were removed from the analysis. Data from phones that were moving within the pocket or bag during the test were also removed from the analysis due to the acceleration noise introduced by this movement. Data duration shorter than 3.50 seconds was considered as early termination of the Gait and Balance test and removed from the analysis. Step length was calculated by the distance and step count collected by the pedometer. A total of 87 gait features were generated by an open-source tool, mhealthtools [21], which decomposes the acceleration data into more sophisticated summary indicators using Fourier Transforms.

In the 9-Hole Peg Test, patients were asked to place a virtual peg in a virtual hole and then remove the virtual peg from the virtual hole as quickly as possible on their phone screen. For patient convenience, this task was repeated 4 times instead of the typical 9 times. The time required to complete the assessment and the total distance the peg was moved during the assessment were collected. Hand speed was calculated by dividing the distance by the time. Sixty hand features were generated, including statistical summaries such as means, medians, minimums, and maximums of dominant and subordinate hand movement of placing and removing the peg.

### Structured Interviews

After completion, the patients were interviewed about issues they experienced with the app. The structured interview (Table 1) included 4 topics: enrollment and app downloading, informed consent forms, demographics and PRO questionnaires, and functional assessments.

**Table 1:** The structured interview guides.

Topics	Questions
Enrollment and app downloading	1. What challenges, if any, did you experience in enrolling in the study?
Informed consent forms	2. Did you experience any difficulty in reading and understanding the informed consent forms?
Demographics and patient-reported outcome questionnaires	3. Which study questions, if any, were difficult to answer? Why?
Functional assessments	4. Which functional assessments, if any, were difficult or uncomfortable to perform? Why?

### Statistical Analysis

Patients were classified as CIPN cases if they reported “quite a bit” or “very much” for at least one of the first 4 questions of the CIPN20 questionnaire [8]. The first 4 questions ask about numbness or tingling in the feet or hands, which are the most common symptoms of CIPN [22,23]. This shortened version has been used to screen long-term CIPN in a large clinical trial [5]. Patients that did not complete CIPN20 within NeuroDetect were classified based on their answers during screening. To test the feasibility of functional assessments via a smartphone app for detecting CIPN, task features available in at least 70% of patients were compared between CIPN case and control groups. Additionally, alternative CIPN case classifications were explored by classifying patients as CIPN feet cases if they responded 3 or more on either of the 2 sensory CIPN feet questions within the CIPN2Feet. A similar approach was used to classify patients as CIPN hands cases. In all instances, any patients not included in the CIPN case group were included in the control group for comparisons. Exploratory analyses of secondary endpoints of cumulative CIPN20 score and PRO-CTCAE severity and interference were also conducted. Principal component analysis (PCA) was conducted using the prcomp function with scaling. Partial least squares discriminant analysis (PLSDA) and PLS regression analysis were conducted using the plsr function in pls package with the orthogonal scoring method and scaling. Features from PLS models with loadings ≥0.2 or ≤–0.2 or variable importance in projection (VIP) score ≥2 were considered important. Predictive performance of PLS models were evaluated with leave-one-out validation. Important features were tested with unpaired 2-sample *t* tests with uncorrected α=.05. Differences in demographics and patient-reported CIPN between CIPN cases and controls were examined by unpaired 2-sample *t* tests and chi-square tests with α=.05. All statistical analysis and visualization were done in R 3.6.3 (R Foundation for Statistical Computing).

## Results

### Enrolled Patients

A total of 26 patients who had completed neurotoxic chemotherapy enrolled and participated in the NeuroDetect assessments. Patients were classified based on the 4 CIPN20 questions. There were 2 patients that did not complete CIPN20, and they were classified as CIPN cases based on their answers during screening. A total of 17 CIPN cases and 9 controls were enrolled. CIPN cases were nominally older (54 vs 49) and completed chemotherapy more recently (2.53 vs 8.27 months), but none of the clinical factors were significantly different between groups (Table 2).

**Table 2:** Clinical data of patients included in the analysis (no variables were significantly different between the 2 groups).

Demographics			Controls (n=9)		Cases (n=17)		*P* value
Age (years), mean (SD)			49 (12.4)		54 (8.2)		.96
**Race, n (%)**							.66
	White	8 (89)		14 (82)			
	Other	1 (11)		3 (18)			
Height (m), mean (SD)			1.78 (0.14)		1.70 (0.10)		.81
Weight (kg), mean (SD)			83.1 (23.6)		75.3 (16.6)		.27
**Cancer type, n (%)**							.59^a^
	Breast	4 (44)		5 (29)			
	Ovarian	0 (0)		2 (12)			
	Lung	0 (0)		1 (6)			
	Colorectal	2 (22)		6 (35)			
	Other^b^	4 (44)		8 (47)			
**Neurotoxic** **chemotherapy agent** **, n (%)**							.60
	Taxane	5 (56)		6 (35)			
	Platinum	2 (22)		5 (29)			
	Taxane and platinum	2 (22)		6 (35)			
Time since treatment completion (months), mean (SD)			8.27 (16.7)		2.53 (2.70)		.08

^a^Chi-square test between chemotherapy-induced peripheral neuropathy cases and controls that had breast cancer, ovarian cancer, lung cancer, or colorectal cancer versus only other cancer types.

^b^Other cancer types include liver cancer, esophageal cancer, prostate cancer, cervical cancer, and pancreatic cancer.

### Patient-Reported CIPN

The median CIPN20 scores were higher in CIPN cases than in controls (28.1 vs 10.0, *P*<.001; Table 3), which was implied by the grouping criteria. All of the CIPN20 subscale scores were also higher in CIPN cases, including CIPN8 (*P*<.001), CIPN4 (*P*<.001), CIPN2Feet (*P*<.001), CIPN2Hands (*P*=.004), and CIPNM (*P*=.02). The median PRO-CTCAE severity and interference were higher in cases (2 vs 3, *P*<.001 and 1 vs 2, *P*<.001, respectively). Only 1 CIPN case reported having fallen since the last chemotherapy session.

**Table 3:** Patient-reported CIPN.

Scale		Controls (n=9)	Cases (n=17)	Feet cases (n=13)	Hands cases (n=9)	
**CIPN20^a^, median (SD)^b^**						
	CIPN20	10.0 (8.8)	28.1 (17.0)	29.8 (16.5)	43.3 (18.6)	
	CIPN8 (Sensory)	10.4 (12.1)	35.4 (21.3)	37.5 (20.8)	58.3 (23.0)	
	CIPNM (Motor)	8.3 (11.0)	20.9 (19.4)	25.0 (19.5)	33.3 (20.5)	
**PRO-CTCAE^c^, median (SD)^d^**						
	Severity	2 (0.7)	3 (0.8)	3 (0.8)	3 (0.8)	
	Interference	1 (0.3)	2 (0.8)	2 (0.8)	2 (1.0)	
Patients reporting a fall, n (%)		0 (0)	1 (6)	1 (8)	0 (0)	

^a^CIPN20: European Organization for Research and Treatment of Cancer Quality of Life Questionnaire for Chemotherapy-induced Peripheral Neuropathy 20-item scale.

^b^There were 2 cases that did not complete CIPN20.

^c^PRO-CTCAE: Patient-Reported Outcomes version of the Common Terminology Criteria for Adverse Events.

^d^There were 3 cases that did not complete PRO-CTCAE.

### Functional CIPN Analysis

**Figure 1:**
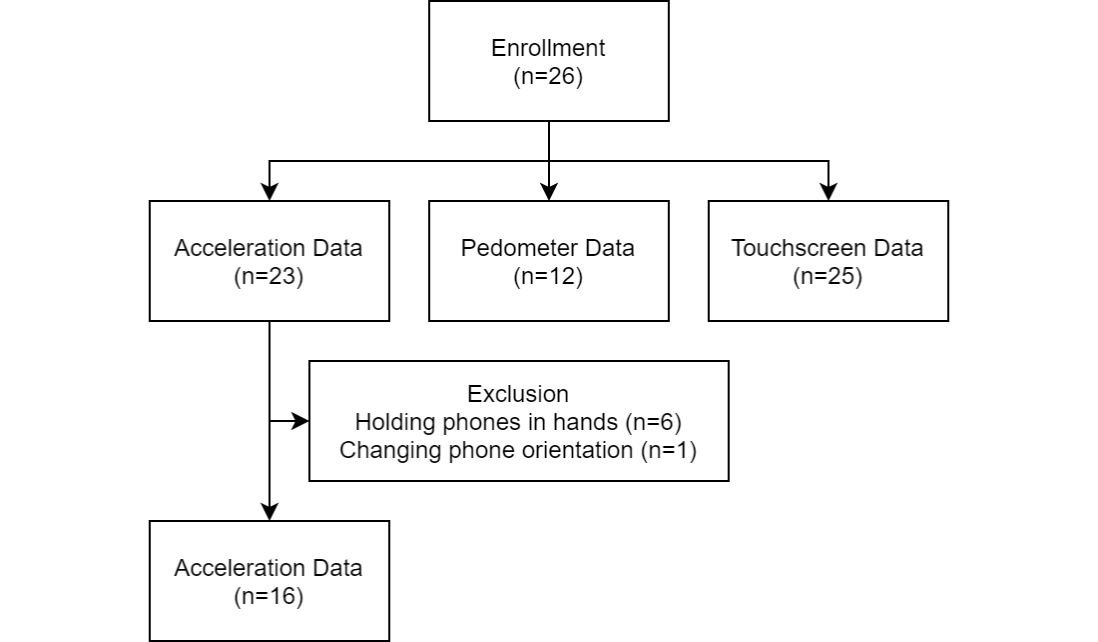
Sample size of each type of data. Excluded acceleration data were from 5 CIPN cases and 2 controls.

**Figure 2:**
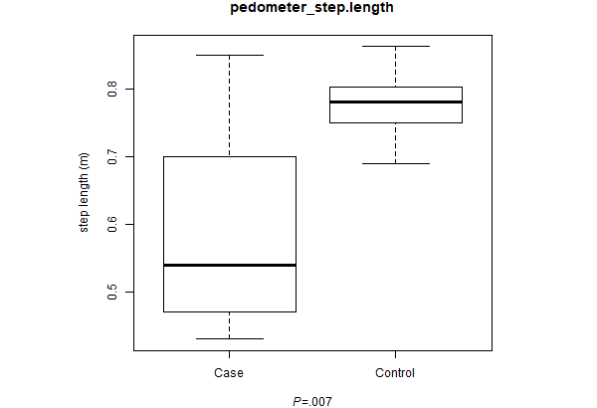
Step length was calculated by the distance and step count collected by the pedometer. The median step lengths were shorter in CIPN cases than controls (0.54 vs 0.78 m, *P*=.007).

Twelve patients gave NeuroDetect permission to collect pedometer data during the Gait and Balance test (Figure 1). The median step lengths were shorter in cases compared with controls (0.54 vs 0.78 m; *P*=.007; Figure 2).

**Figure 3:**
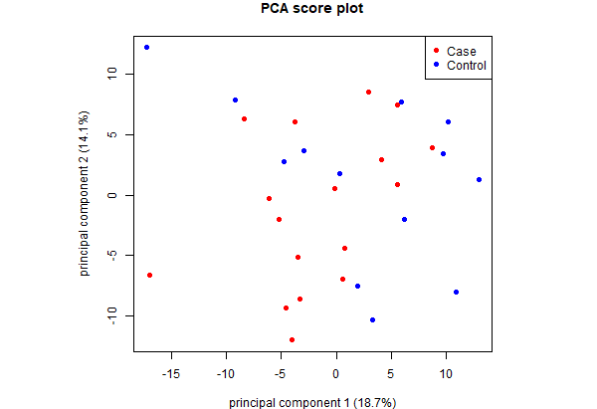
Score plot of PCA integrating mhealthtools gait features and hand features. The first 2 components explained 32.8% of the variance between CIPN cases and controls. However, no individual features showed high importance in the first 2 components (all loadings between -0.2 and 0.2). CIPN: chemotherapy-induced peripheral neuropathy; PCA: principal component analysis.

Acceleration data from 7 patients (Figure 1) were removed from the PCA and PLS analyses due to holding the phone in their hands (n=6) or phone changing orientation in the pocket during the test (n=1). Two patients completed only 1 walk of the Gait and Balance test, so only the completed walk was used in the analysis. Using PCA to integrate mhealthtools gait features and hand features of 16 patients, the first 2 components explained 32.8% of the variance between cases and controls (Figure 3), and each of the remaining principal components explained ≤10.0% of the variance. However, no individual features showed high importance (all loadings between –0.2 and 0.2) in the first 2 principal components.

**Figure 4:**
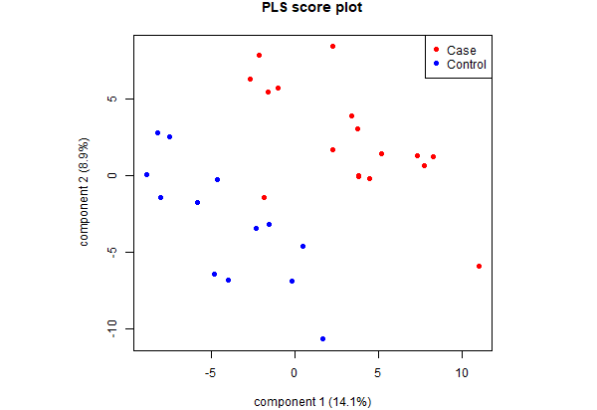
Score plot of partial least squares discriminant analysis integrating mhealthtools gait features and hand features. The first 2 components achieved good separation between CIPN cases and controls and explained 23.0% of the variance between groups. In the first 2 components, 145 features had variable importance in projection scores ≥1, including 12 with scores ≥2 (see Figure 5). All loadings between -0.2 and 0.2. CIPN: chemotherapy-induced peripheral neuropathy; PLS: partial least squares.

**Figure 5:**
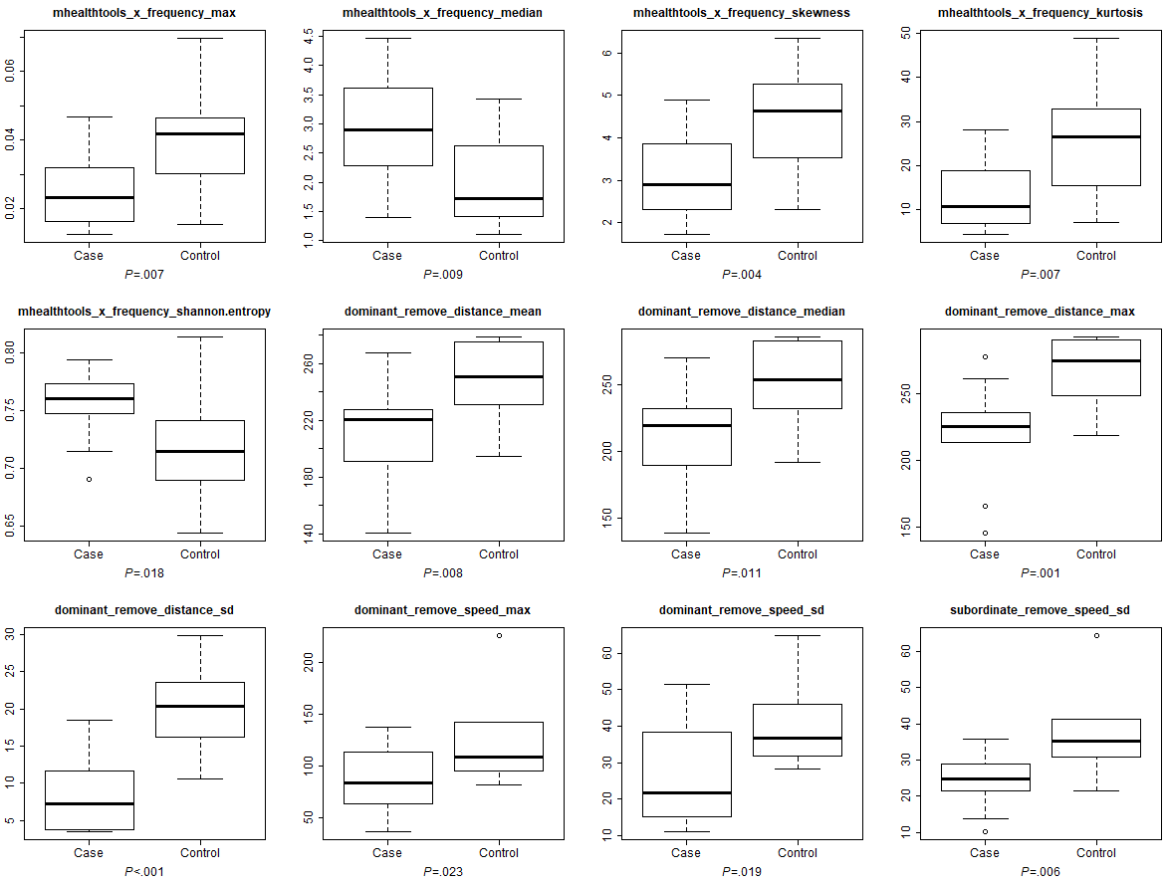
A total of 12 important features with variable importance in projection scores ≥2 in the first 2 components of partial least squares discriminant analysis. The *P* values annotated in each subplot were from unpaired two-sample t tests. The x-axis is the swaying axis. CIPN cases had higher Shannon entropy (*P*=.02), higher median frequency (*P*=.009), lower maximum frequency (*P*=.007), lower skewness (*P*=.004), and lower kurtosis (*P*=.007) in the swaying axis. CIPN cases also had lower mean (*P*=.008), median (*P*=.01), maximum (*P*=.001), and SD (*P*<.001) of the distance and lower maximum (*P*=.02) and SD (*P*=.02) of the speed of the peg removing movement in the dominant hands, and lower peg removing speed SD (*P*=.006) in the subordinate hands. CIPN: chemotherapy-induced peripheral neuropathy.

In PLSDA, the first 2 components achieved good separation between CIPN cases and controls (Figure 4). A total of 145 features had VIP scores ≥1 (Multimedia Appendix 1), and 12 features had VIP scores ≥2 (Figure 5). These 12 features were all significant in unpaired 2-sample *t* tests. They were 5 gait features in the swaying axis and 7 hand features. In terms of gait features of frequency decomposition in the swaying axis, CIPN cases had higher Shannon entropy (*P*=.01), higher median frequency (*P*=.009), lower maximum frequency (*P*=.007), lower skewness (*P*=.004), and lower kurtosis (*P*=.007). In terms of hand features, CIPN cases had lower mean (*P*=.008), median (*P*=.01), maximum (*P*=.001), and SD (*P*<.001) of the distance, and lower maximum (*P*=.02) and SD (*P*=.01) of the speed of the peg removing movement in the dominant hands, and lower peg removing speed SD in the subordinate hands (*P*=.006). The first 6 components explained 52.4% of the variance between groups, while each of the remaining components explained ≤6.1% of the variance. The leave-one-out validation of the first 6 components in the PLSDA model showed *R*^2^=0.590 and RMSE=0.312 (Multimedia Appendix 2).

Exploratory PLSDA classifying patients as CIPN feet cases identified the same set of features, and the model of CIPN hands cases identified a similar set of the peg removing distance features in the dominant hands and additional gait features with high VIP scores (VIP>2; additional gait features are shown in Multimedia Appendix 3).

Exploratory PLS regression analyses of the secondary endpoints of cumulative CIPN20 score and PRO-CTCAE severity and interference identified several of the same features that were identified in the prior case–control analyses, including gait features in the swaying axis and hand features of the peg removing movement in the dominant hands (additional features are shown in Multimedia Appendix 4).

### Structured Interviews

Among the 26 patients, 12 cases and 7 controls participated in the interviews. Of these, 5 patients (26%) experienced challenges during the enrollment due to the password requirement. Two (11%) had problems reading the consent form and preferred the format to be a continuous scroll, while it was a booklet format in the app where a user clicks to navigate to the next page. A total of 11 patients (58%) had issues with the questionnaires, and among them, 8 patients wished they had an option to go back and change their answers to the baseline demographic questions, and 3 patients found it difficult to measure their neuropathy symptoms in the scale from 1 (not at all) to 4 (very much). Four (21%) had issues with the Gait and Balance test because they were too tired or did not feel comfortable performing the test. Four patients (21%) had difficulty understanding the 9-Hole Peg Test.

## Discussion

### Principal Findings

CIPN is a common and debilitating toxicity of chemotherapy that has both subjective and objective components. Smartphone apps are being used to collect subjective data via PRO and may also be a convenient platform for integrating objective CIPN assessments. Our pilot study supported the feasibility of downloading and completing remote assessments within a smartphone app and explored whether these functional assessments could differentiate patients with and without CIPN. We found evidence that patients with CIPN have shorter step length, unique swaying acceleration patterns during a walking task, and shorter hand moving distance in the dominant hands during a manual dexterity task. Our results indicate that functional assessments of feet and hands via a smartphone app may be helpful in detecting CIPN during chemotherapy treatment, but future studies are needed.

### Feasibility and Adherence

As described above, among the patients enrolled in this study, 2 cases did not complete CIPN20, and 3 cases did not complete PRO-CTCAE (Table 2). Over half did not allow the collection of pedometer data, and 7 did not follow instructions to place their phone in their pocket during the walking task (Figure 1). These nonadherence issues led to some loss of data. The interview results showed that some patients had difficulties using the app due to the password requirements, demographics questionnaire, and functional assessments, which indicated a need for additional training or oversight during the initial app download.

### Comparison With Prior Work

Shorter step length, slower gait speed, and loss of balance are the most common CIPN symptoms, and these symptoms progress throughout treatments [4,24,25]. In patients with CIPN, wearable sensors can detect impairment in stride length, stride duration, and gait speed during a natural walk, and an increase in sway when standing [10,11]. However, a prior study in patients that self-reported peripheral neuropathy symptoms caused by exposure to chemotherapy found no correlation between CIPN severity and a single posttreatment assessment of gait and balance, including stride time, cadence, speed, or sway [26]. Our exploratory study found that CIPN cases had shorter step lengths. Using the frequency decomposition methods in mhealthtools, we also found that the frequency spectrum of CIPN cases acceleration data had higher median frequency and lower maximum frequency and were more symmetrical and less predictable. These differences in frequency distribution could indicate CIPN symptoms, even if they do not have an intuitive interpretation. Our seemingly discordant findings may be due to advances in collecting and analyzing gait and balance data via the smartphone app. Additional work is needed to identify the specific gait features most strongly indicative of CIPN, similar to the development of algorithms for detecting Parkinson disease using mPower data [21,27,28]. Future work should also consider alternatives to a natural walk test, including balance assessments commonly used during clinical neurological assessments such as walking heel-to-toe (tandem walk) [29] or balancing with your eyes closed (Romberg test) [30].

In the 9-Hole Peg Test, we found that CIPN cases had shorter distance and lower distance SD of the peg removing movement in the dominant hands. We also found slower maximum peg removing speed in the dominant hands and lower peg removing speed SD in both hands. Motor weakness is a common manifestation of CIPN [2,3]. A hand kinematic analysis in patients that reported numbness with or without neuropathic pain due to chemotherapy used 3D recordings of the hand grip-release test and revealed CIPN cases had more jerks in grasp movement but not in reach movement [31]. It would be difficult to adapt this test into a smartphone app–based functional assessment, but there may be opportunities for other manual strength and dexterity tasks such as finger tapping [32,33] or line tracing [34].

### Impact on Patient Care

Our pilot study indicates that a smartphone app–based functional assessment may be helpful in detecting CIPN and could possibly be useful for CIPN monitoring during neurotoxic chemotherapy treatment. Because CIPN can manifest in the feet or hands [2,3], our smartphone app includes both gait and hand assessments. CIPN monitoring via a smartphone would circumvent several challenges to objective CIPN assessment. Patients could complete assessments whenever and wherever is most comfortable and convenient for them. Collection via a smartphone does not require any trained personnel or specialized equipment other than a free app, eliminating most costs associated with CIPN monitoring. The seamless integration of subjective and objective CIPN data collection within a smartphone app may improve early CIPN detection, allowing for early initiation of treatment modification to prevent irreversible, life-altering toxicity [35].

### Limitations

This is the first study that identified smartphone sensor gait features associated with CIPN, and this is also the first study using a smartphone app–based functional assessment for hand symptoms in CIPN. Despite these strengths, this study has several limitations that are worth considering. First, this was a small study of a heterogeneous cohort of patients and a large number of variables. Because of the exploratory nature of this study, we allowed a higher false-positive rate and did not correct for multiple comparisons in unpaired 2-sample *t* tests. The 2 smartphone app–based functional assessments and the open-source data decomposition tool have not been tested in patients with CIPN. Larger studies are needed to validate the smartphone app–based tests and features indicative of CIPN and adjust for confounders such as comorbidities. Second, another major limitation was the assessment at a single time point after completion of chemotherapy. There may be differences between patient’s willingness or ability to complete the app-based assessment after treatment versus during treatment. Our subsequent study will collect assessments before and during treatment to determine the feasibility of assessment during treatment and to better understand the functional changes caused by CIPN. Third, the smartphone app–based functional assessments were affected by users’ consent to data collection and adherence to the app instruction. In future studies, we will need more specific instructions during the initial app downloading to ensure that valid data are collected from all users. Finally, smartphone app–based gait analyses are more reliable if the phone is placed at the lumbar location [27]. Our data may have more variability caused by the placement of devices in a pocket or bag, but this is a trade-off we have made to develop an app that requires no external equipment, including a harness or wearable sensors.

### Conclusions

Our findings suggest that smartphone app–based functional assessments may be useful to detect functional impairment indicative of CIPN. Future work will conduct longitudinal assessments in patients undergoing neurotoxic chemotherapy using a second-generation app that includes additional functional assessments to identify features that are most strongly associated with CIPN and determine whether functional assessment detects CIPN prior to a patient’s subjective assessment. Upon validation, we may be able to integrate convenient, low-cost smartphone-based CIPN monitoring into chemotherapy treatment to improve detection and prevent irreversible and life-altering CIPN.

## Appendix

Multimedia Appendix 1 Variable importance in projection score distribution of the first 2 components of partial least squares discriminant analysis. A total of 145 features had variable importance in projection scores ≥1. Among them, 12 had variable importance in projection scores ≥2 (see <xref ref-type="fig" rid="figure5">Figure 5</xref>).

Multimedia Appendix 2 Leave-one-out validation of the first 6 components in partial least squares discriminant analysis. R2 = 0.590 and RMSE = 0.312.

Multimedia Appendix 3 A total of 11 additional gait features with variable importance in projection scores ≥2 were identified in the first 2 components of the exploratory partial least squares discriminant analysis of CIPN hand cases. The <italic>P</italic> values annotated in each subplot were from unpaired two-sample t test. The x-axis is the swaying axis, and the z-axis is the vertical axis. In the vertical axis, CIPN hand cases had higher maximum frequency (<italic>P</italic>=.044), skewness (<italic>P</italic>=.028), kurtosis (<italic>P</italic>=.043), and lower energies in lower frequency bands (band 6 <italic>P</italic>=.014, band 6.5 <italic>P</italic>=.005, band 7 <italic>P</italic>=.007) of frequency decomposition and lower entropies of empirical wavelet transform (Shannon entropy <italic>P</italic>=.049, permutation entropy <italic>P</italic>=.049, Renyi entropy <italic>P</italic>=.029).

Multimedia Appendix 4 A total of 11 additional features with variable importance in projection scores ≥2 were identified in the first 2 components of the exploratory partial least squares regression analysis of CIPN20 and PRO-CTCAE severity and interference. The <italic>P</italic> values annotated in each subplot were from unpaired two-sample t test. The x-axis is the swaying axis, and the z-axis is the vertical axis. Among the gait features of frequency decomposition, higher energy in a lower frequency band in the swaying axis (band 4.5 <italic>P</italic>=.049) and higher energies in medium frequency bands (band 12 <italic>P</italic>=.047 and band 12.5 <italic>P</italic>=.037) were associated with worse CIPN scores. Among the hand features, the maximum peg removing speed in the subordinate hands were associated with worse CIPN scores.

## Abbreviations

CIPN: chemotherapy-induced peripheral neuropathy
CIPN20: European Organization for Research and Treatment of Cancer Quality of Life Questionnaire for Chemotherapy-induced Peripheral Neuropathy 20-item scale
PCA: principal component analysis
PLSDA: partial least squares discriminant analysis
PRO: patient-reported outcome
PRO-CTCAE: Patient-Reported Outcomes version of the Common Terminology Criteria for Adverse Events
VIP: variable importance in projection
