# Supplementary figures and images for: Chemotherapy-Induced Peripheral Neuropathy Detection via a Smartphone App: Cross-sectional Pilot Study
Source: JMIR Mhealth Uhealth. 2021 Jul 5;9(7):e27502. doi: 10.2196/27502 (PMC8406129; doi:10.2196/27502)

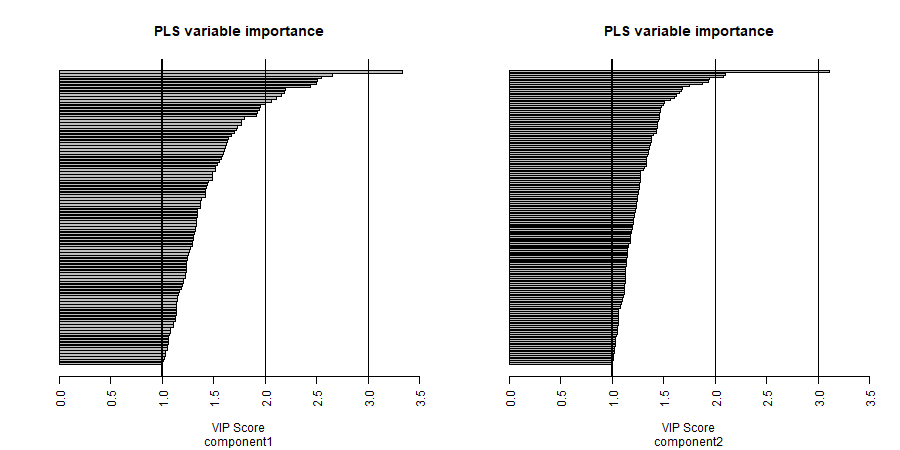

Supplement: Multimedia Appendix 1 [file mhealth_v9i7e27502_app1.png]

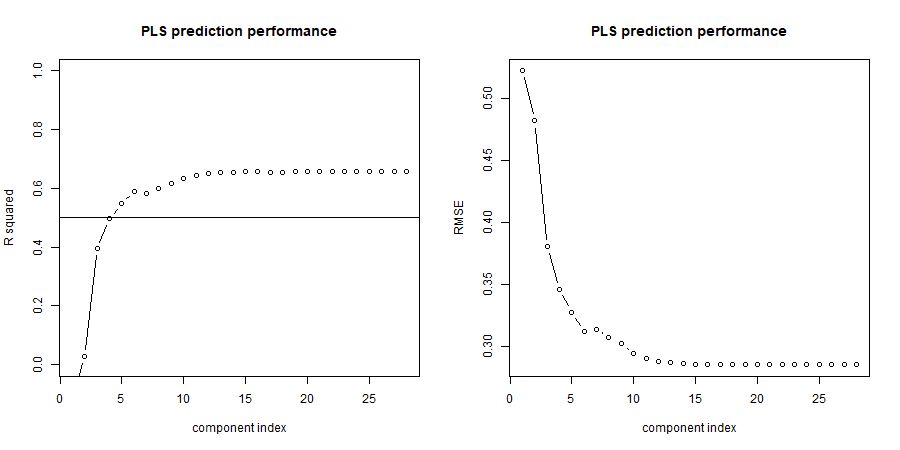

Supplement: Multimedia Appendix 2 [file mhealth_v9i7e27502_app2.png]

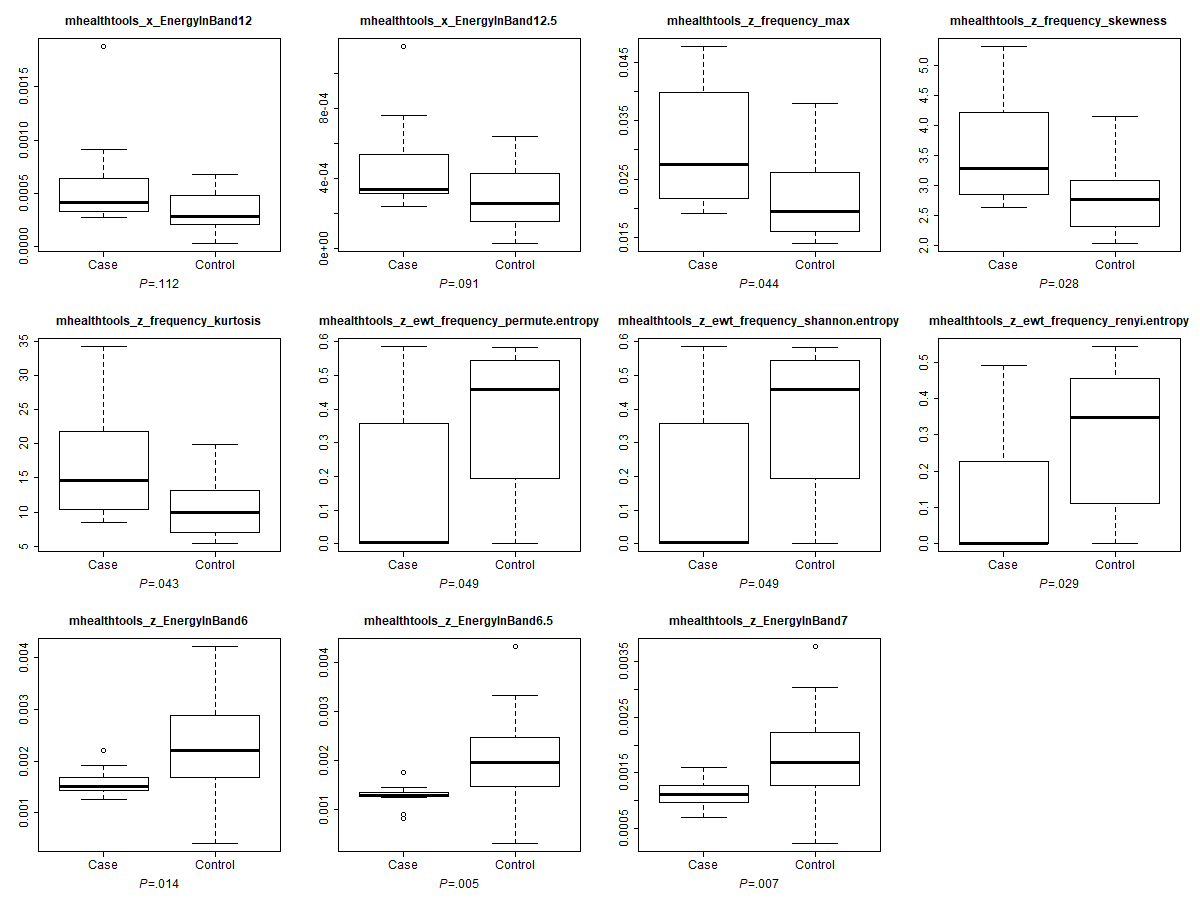

Supplement: Multimedia Appendix 3 [file mhealth_v9i7e27502_app3.png]

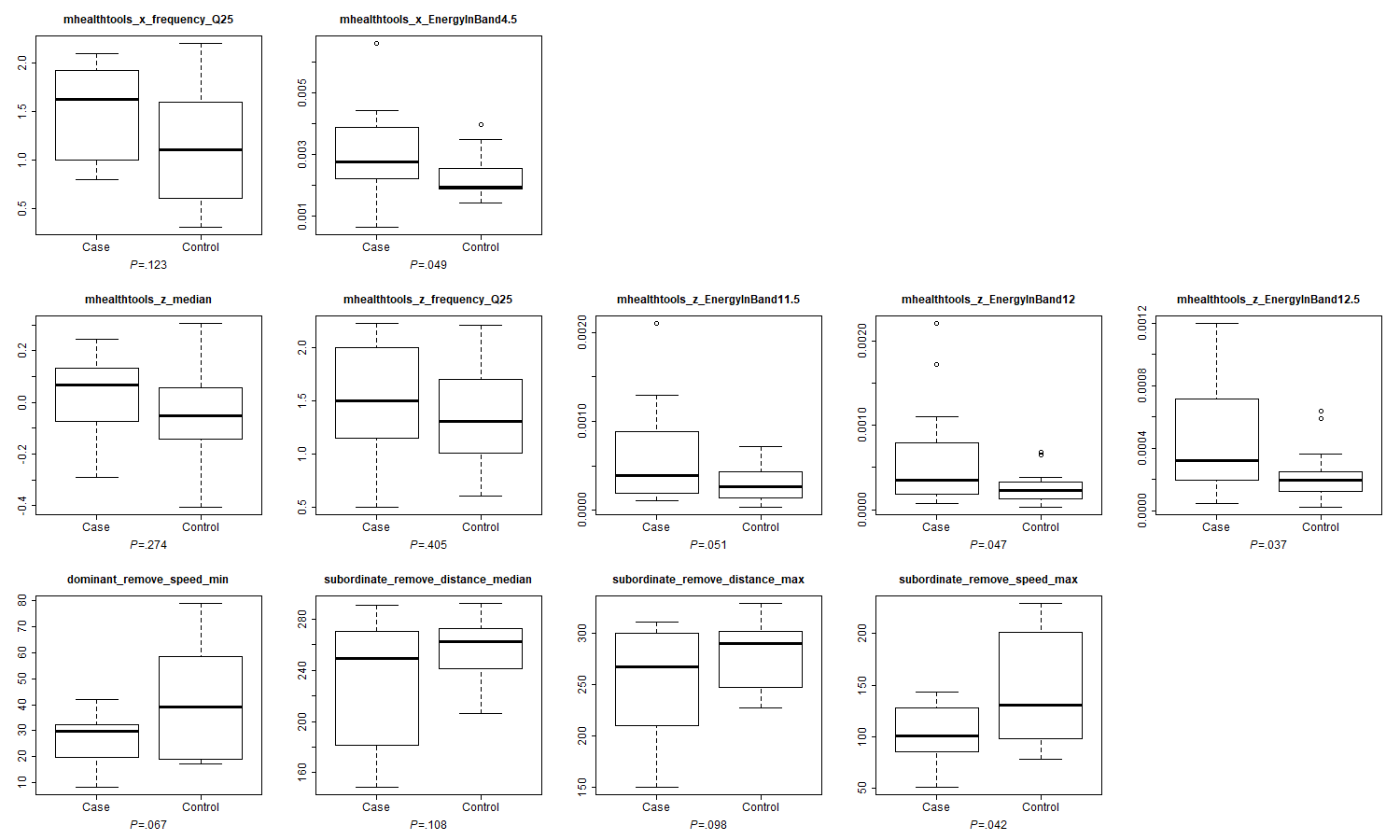

Supplement: Multimedia Appendix 4 [file mhealth_v9i7e27502_app4.png]
